# Linking health survey data with health insurance data: methodology, challenges, opportunities and recommendations for public health research. An experience from the HISlink project in Belgium

**DOI:** 10.1186/s13690-023-01213-0

**Published:** 2023-11-15

**Authors:** Finaba Berete, Stefaan Demarest, Rana Charafeddine, Karin De Ridder, Herman Van Oyen, Wannes Van Hoof, Olivier Bruyère, Johan Van der Heyden

**Affiliations:** 1 Department of Epidemiology and Public Health, Sciensano, Juliette Wytsmanstraat 14, Brussels, 1050 Belgium; 2 Department of Public Health, Epidemiology and Health Economics, University of Liège, Liège, Belgium; 3 Department of Public Health and Primary Care, Ghent University, Ghent, Belgium; 4 WHO Collaborating Centre for Public Health Aspects of Musculoskeletal Health and Ageing, Research Unit in Public Health, Epidemiology and Health Economics, University of Liège, Liège, Belgium

**Keywords:** Record linkage; data linkage, Health administrative insurance data, Health claims data, Health interview surveys

## Abstract

In recent years, the linkage of survey data to health administrative data has increased. This offers new opportunities for research into the use of health services and public health. Building on the HISlink use case, the linkage of Belgian Health Interview Survey (BHIS) data and Belgian Compulsory Health Insurance (BCHI) data, this paper provides an overview of the practical implementation of linking data, the outcomes in terms of a linked dataset and of the studies conducted as well as the lessons learned and recommendations for future links.

Individual BHIS 2013 and 2018 data was linked to BCHI data using the national register number. The overall linkage rate was 92.3% and 94.2% for HISlink 2013 and HISlink 2018, respectively. Linked BHIS-BCHI data were used in validation studies (e.g. self-reported breast cancer screening; chronic diseases, polypharmacy), in policy-driven research (e.g., mediation effect of health literacy in the relationship between socioeconomic status and health related outcomes, and in longitudinal study (e.g. identifying predictors of nursing home admission among older BHIS participants). The linkage of both data sources combines their strengths but does not overcome all weaknesses.

The availability of a national register number was an asset for HISlink. Policy-makers and researchers must take initiatives to find a better balance between the right to privacy of respondents and society’s right to evidence-based information to improve health. Researchers should be aware that the procedures necessary to implement a link may have an impact on the timeliness of their research. Although some aspects of HISlink are specific to the Belgian context, we believe that some lessons learned are useful in an international context, especially for other European Union member states that collect similar data.

**Supplementary Information:**

The online version contains supplementary material available at 10.1186/s13690-023-01213-0.

## Background

An evidence-based health policy requires sound and reliable health data and appropriate research methods from which it can be explored. To answer research questions, researchers can rely both on data derived from health surveys and on administrative data, such as health insurance data, health care data from primary care or hospital information systems, disease-specific registers, etc. [1]. Although administrative data is initially collected for other purposes, it is increasingly being used as a secondary data source for research. Such secondary data is generally easily accessible, resource-efficient and offers additional advantages, depending on the nature and the source [2].

Data linkage brings together information that relates to the same individual, family, place or event from different data sources [3, 4]. Single data sources are more commonly insufficient for answering complex research and policy questions. When answering these questions, the repeated collection of primary data is less flexible, more costly and more complex compared to data linkage. In countries where administrative data linkage is traditionally well established (e.g. in the UK, Australia, Canada, the Nordic countries, etc.), linked data is increasingly used for public health research purposes [5–7]. Internationally, data linkage is common and an accepted practice for population health research and monitoring [8], especially to leverage existing data. Indeed, data linkage is a powerful and a cost-effective method for cohort studies. For example, in Germany, the lidA- leben in der Arbeit is a cohort study on work, age and health which uses survey data that is linked to claims data from a large amount of statutory health insurance data [9]. Furthermore, such data linkage is a well-established method for external validations. Surveys data may be subject to bias (selection bias, recall bias) or may be inaccurate. Data linkage is a useful tool to validate such information. For instance, Hall et al. studied the validity of self-reported screening for prostate cancer and colorectal cancer in the United States [10]. Van der Heyden et al. (2016) also assessed the validity of self-reported information on health care use [11]. In another study, the same author estimated the predictive validity of the Global Activity Limitation Indicator (GALI) in the general population in Belgium [12].

In Belgium, the Belgian Health Interview Survey (BHIS) and the Belgian Compulsory Health Insurance (BCHI) are important sources of information on population health and healthcare consumption and are complementary. The National Institute for Health and Disability Insurance (NIHDI) commissioned a linkage study between BHIS and BCHI data with 3 specific questions: (1) to explore regional differences in healthcare consumption in more depth; (2) to assess the validity of healthcareconsumptionbased chronic disease indicators; (3) to estimate the cost to Belgian health insurance if some groups of non-reimbursed medicines (analgesics, laxatives and calcium supplements) were to be reimbursed [13]. Moreover, the linked data was used in further studies [11, 12). The HISlink project was then launched in 2017 as a systematic linkage between each wave of BHIS and BCHI data.

Linking BHIS and BCHI data sources allows the strengths of different data sources to be used synergistically and provides opportunities for new and advanced research. While BHIS data on medical consumption may be subject to recall bias, may be inaccurate and are prone to substitution by BCHI data, it is a source for detailed information on sociodemographic data, health-related behaviour and mental health. BCHI data also addresses elements that cannot be collected by means of a survey (e.g., healthcare expenditure, medical procedures).

While linkage of administrative-to-administrative data has a long tradition [9, 14–19], linkage of survey data with administrative data is a relatively new field with great potential [9] and with its own challenges and considerations to take into account. These challenges may vary according to the context and the applicable data protection requirements. However, there is a paucity of information on the research opportunities and challenges faced when linking survey and administrative data. This study aims to fill these gaps.

Within the framework of HISlink, data from two BHIS waves has been linked to BCHI data: the BHIS2013 and BHIS2018. Using the case of these two linkages, this paper aims to discuss the methodology and the lessons on barriers and opportunities of linking survey data with health insurance data. More specifically, the focus will be on the following items: the practical implementation and outcomes in terms of linked datasets and the studies conducted, lessons learned and recommendations for future linkages. Although the Belgian context may be different from those of other countries, we believe that such information could be relevant for future researchers who plan to link surveys and health insurance data.

## The implementation of individual data linkage: an experience based on the HISlink study

In Belgium, the BHIS and the BCHI have been linked for the last three waves of the BHIS, conducted in 2008 (as part of a feasibility study), 2013 and 2018. At the time of writing, the BHIS2008 data link had been destroyed due to the expiry of the retention period. Therefore, in this study, only the linkage of BHIS2013 and BHIS2018 are considered. This section describes the data sources, the linkage process and the privacy issues that arose and how they were overcome.

### Description of data sources

HISlink combines BHIS and BCHI data sources. An overview of the most essential features of HISlink database is displayed in Table S1 (Supplementary file).

#### BHIS data

The BHIS is a national, cross-sectional household survey conducted every 5 years since 1997 by Sciensano, the Belgian health institute, among a representative sample of Belgian residents, including older, institutionalized people. Participants are selected from the national population register, using a multistage, stratified-sampling design [20]. The participation rate of the survey at a household level was 57.1% and 57.5% for BHIS2013 and BHIS2018 respectively. Information is collected through a Computer-Assisted Personal Interview (CAPI) and a paper and pencil questionnaire for the more sensitive questions. Detailed methodology of the survey can be found in Demarest et al. (2013) [20]. Though BHIS has several advantages: data are collected at a total population level, including people who do not make use of health services. Information is obtained from the perspective of the individual him/herself. The collection of self-perceived health, lifestyle and behaviour data is only (or mainly) possible through a survey. Information is collected simultaneously on individuals’ health status, health behaviour and the use of health care, but also on socio-demographic health determinants, such as socio-economic status. This horizontal data collection makes it possible to study the relationship between different domains and topics. The different waves of BHIS enables trends analysis [21]. However, as with all surveys, the organization of BHIS is expensive and time-consuming. Moreover, BHIS data is self-reported and therefore subject to biases such as selection bias, recall bias or social-desirability bias [9, 11, 22, 23]. For instance, BHIS data on medical consumption may be subject to recall bias, may be inaccurate and prone to substitution by BCHI data (i.e. objective health-consumption data).

#### BCHI data

In Belgium, there is compulsory health insurance which is a source of exhaustive and detailed data on the reimbursed health expenses of almost 99% of the total population. However, there are some differences in coverage rates between regions and demographic characteristics [24]. Since 2002, the InterMutualistic Agency (IMA), an overarching national organisation, collects and manages data on all Belgian citizens from these sickness funds (hereinafter referred to as BCHI data). The BCHI database is a longitudinal linkage between 3 components: the individual’s background information, healthconsumption data and database on use of outpatient medicines, which are linked using a Trusted Third Party (TTP) [25, 26], i.e. the linkage was outsourced to another organisation that has access to identifiable data and has performed the linkage. The database includes an arbitrary id-code, allocated by the TTP. The primary goal of the BCHI data is for reimbursement purposes. BCHI data is widely used by important actors in the health field for reimbursementrelated studies, assessment and planning of health care costs. In addition, BCHI data is also used for specific studies beyond its initial intended use (secondary use). One advantage is that the data is not self-reported, nor is it limited to a certain registration period, since there is continuous data collection for administrative purposes. Although BCHI data does not include information on the diagnosis, algorithms have been developed to estimate the prevalence of certain chronic diseases at a general-population level (pseudo pathologies derived from medication use) (27). Furthermore, this enables trend analyses and longitudinal studies [28, 29). BCHI data has some shortcomings: the main limitation of the BCHI is that it only includes information on covered health services and goods, and there is a limited information on outpatient supplements. Next, since the purpose of BCHI data is the billing of services, the data may be subject to errors (e.g. inaccurate procedure codes, upcoding errors, duplicate billing) (30). Detailed information on BCHI data can be found elsewhere [31].

The above description shows that some information is only available in the BHIS (e.g. health status, health behaviour), other information is common to both data sources, even if conceptually different (e.g. health care utilisation, use of medication, as well as a limited amount of socio-demographic information), while other information is only available in the administrative database (specific procedure codes such as nursing home admission, healthcare costs), which therefore makes the two databases complementary. The HISlink 2013 and 2018 resulted in datasets containing around 1200 variables and related indicators from BHIS and 130 variables or indicators from BCHI. Table S1 in supplementary file presents an overview of the content of the linked database, organised by modules, i.e. a set of information related to the same topic.

### The partners involved, the linkage process and data flow

**Fig. 1:**
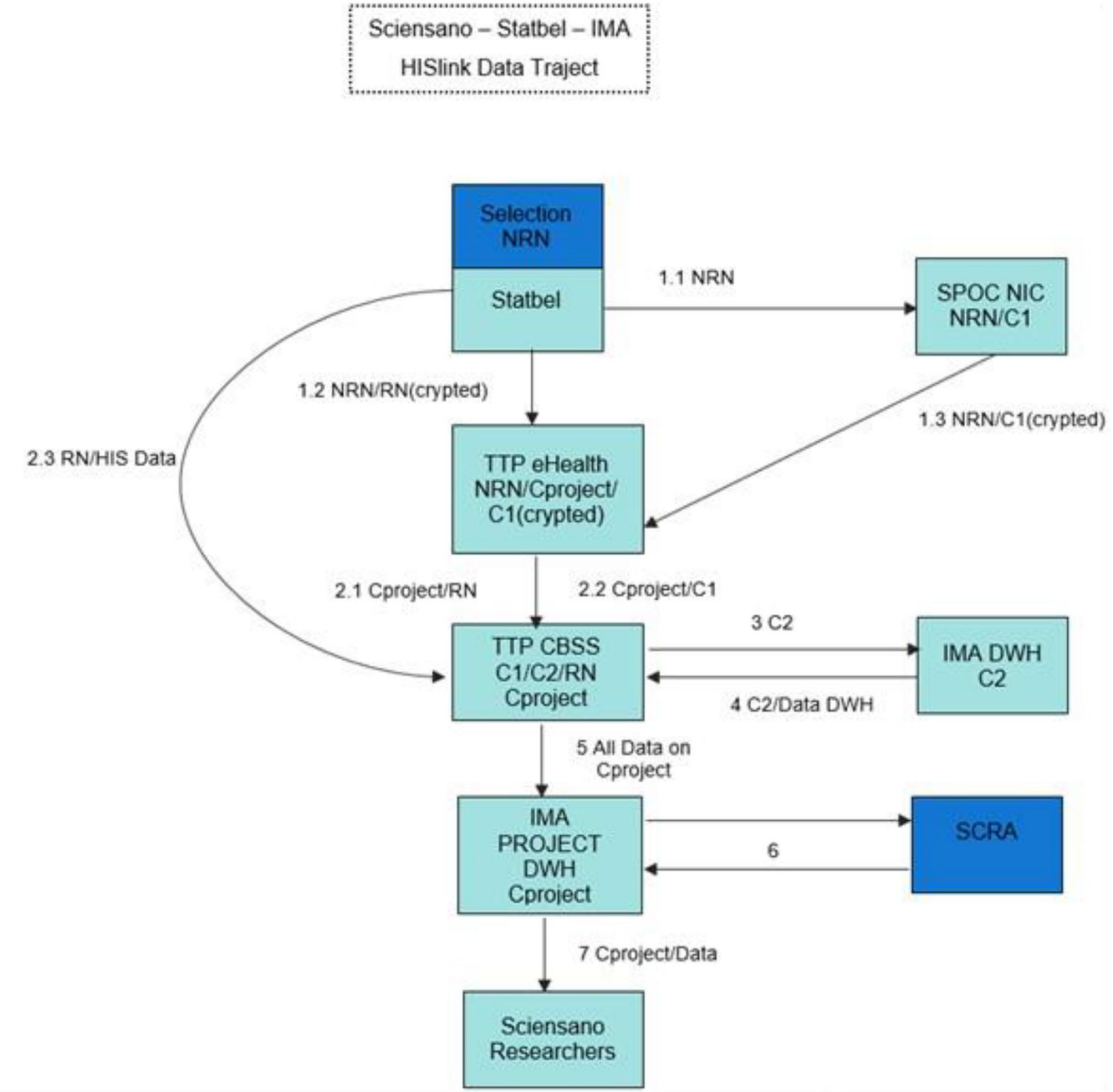
Step-by-step overview of linkage procedure and data coding system to enable data linkage for the HISlink 2018, Belgium NRN: National Register Number; Statbel: the Belgian statistical office; RN: Random Number; SPOC NIC: Single Point of Contact National InterMutualistic College; TTP CBSS: Trusted Third Party Crossroads Bank for Social Security; IMA DWH: InterMutualist Agency Data Warehouse; TTP eHealth: Trusted Third Party eHealth; SCRA: Small Cells Risk Analysis; C1/C2: coding 1/2; Cproject: project specific coding Explanatory note: the link involved the following steps, Fig. 1 1. Statbel selects the NRN of BHIS participants and transmits this selection of NRN to the NIC (1.1) and the selection of NRN with an internal RN (Random Number) specific to this project to the TTP eHealth (1.2). The NIC Security Advisor transmits an NRN/C1-encoded list of persons to the TTP eHealth, with C1 encrypted (1.3) 2. The TTP eHealth sends via the secure eHbox Cproject/RN to the TTP CBSS (2.1). The TTP eHealth sends via the Cproject/C1 secure eHbox to the TTP VI (2.2). Statbel transmits BHIS data on an NR basis to the TTP VI (CBSS° (2.3) 3. On the basis of a second coding (C1 → C2), the data are selected from the IMA DWH (3) 4. The data is sent back on a C2 basis to the TTP CBSS (4) 5. TTP CBSS replaces C2 with Cproject and also converts the received data into Cproject. These are transmitted to the IMA DWH (5) 6. A small cell risk analysis (SCRA) is carried out by the IMA (6) 7. The data sets are made available to Sciensano researchers (Cproject) (7)

Figure 1 presents the data flow and the partners involved at each step. BHIS data is linked at an individual level to BCHI data, using the unique identifier: the national register number (NRN). The linkage is initially done by the reference person. At a later stage, household composition was compared according to BHIS and BCHI information and (based on date of birth, sex and date of the interview) the other household members’ NRN were retrieved. The linkage process is quite complex since it requires several coding processes to ensure privacy and data protection. Detailed information on the linkage process and data flow is provided elsewhere [32]. For the sake of clarity, the linkage scheme has been altered slightly. In summary, during the process, encrypted data are exchanged between the partners in a secure manner. For privacy reasons, there is need to ensure that none of the involved parties would have access to both the sensitive data and the NRNs during the linkage procedure. A small cell risk analysis (SCRA) is carried out by IMA. Only pseudomised data sets are then made available to Sciensano researchers on IMA server. Researchers have access to linked database through a Virtual Private Network (VPN) connection with secure token. Ultimately, a quadruple coding system ensures a coded database where no single party holds all of the respective keys enabling identification of individual patients.

According to the GDPR, the processing of sensitive personal data, such as data concerning health shall be prohibited. However, processing for research is included as one of the exemptions of this rule under certains conditions. Article 5 of the GDPR defines some basic principles that must be taken into account when processing personal data (lawfulness, proportionality, accuracy, data minimization, storage limitation and integrity and confidentiality). The principle of proportionality means that researchers may only process personal data for the purpose of their research, and the processing must be reasonable and proportionate to the purpose of the research. Therefore, proportionality requires data minimisation, meaning that only that personal data which is adequate and relevant for the purposes of the processing is collected and processed [33, 34].

Because of the proportionality principle, only a select amount of information from BHIS and BCHI data is included in the HISlink. An overview of BHIS and BCHI data included in the HISlink can be found in Table S1 (supplementary file). BCHI data covering the period from 2012 (or from 2008 in some specific cases such as dental care or cancer screening) to 2018 (or HISlink2013); and covering the period from 2017 (or from 2013 in some specific cases such as dental care or cancer screening) to 2023 (or HISlink 2018) is included in this study.

### Privacy procedures

The BHIS2013 and BHIS2018 were carried out in line with the Belgian privacy legislation and have been approved by the Ghent University Hospital ethics committee on October 1, 2012 (opinion EC UZG 2012/658) and December 21, 2017 (opinion EC UZG 2017/1454) respectively. Participation in the BHIS is voluntary. No written consent was foreseen. Participation was equivalent to giving consent.

For the linkage to BCHI data, authorization was obtained from the Belgian Information security committee acting as an institutional review board (IRB) (local reference: Deliberation No. 17/119 of December 19, 2017, amended on September 3, 2019, for the HISlink 2013 and local reference: Deliberation No. 20/204 of November 3, 2020 for the HISlink 2018). In its deliberation, the IRB required Sciensano to inform the BHIS participants about the linkage of their data. In view of the disproportionate effort this would require (almost 11,000 individuals for the BHIS2013 and more than 12,000 individuals for the BHIS2018), and since the linkage process was launched before the implementation of the GDPR, Sciensano presented an alternative approach to the IRB, which was accepted. This approach consisted of an exemption from obligation to provide information at an individual level, as well as communication about the data processing, provided to the general public, through a publication on the BHIS website.

### Study population, linkage rates and an evaluation of linkage quality

All BHIS participants were eligible for inclusion in the HISlink. Figure 2a and 2b present the selection process for the final participants: BHIS2013 and BHIS2018, respectively. Overall, the linkage rate was 92.3% for BHIS2013 and 94.2% for BHIS2018.

**Fig. 2a:**
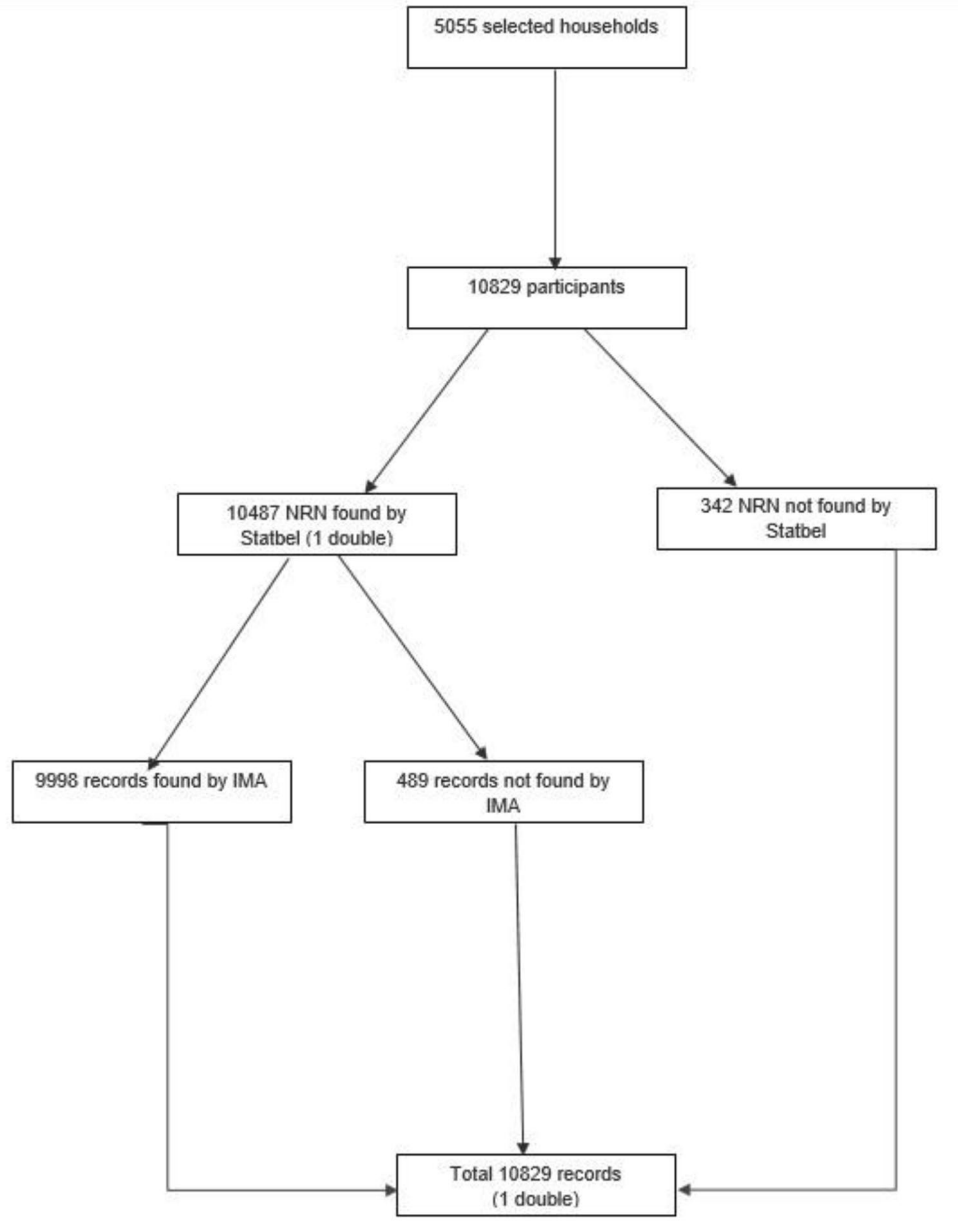
Data flow and linkage global results, HISlink 2013, Belgium *IMA: InterMutualist Agency, NRN: National Register Number*

**Fig. 2b:**
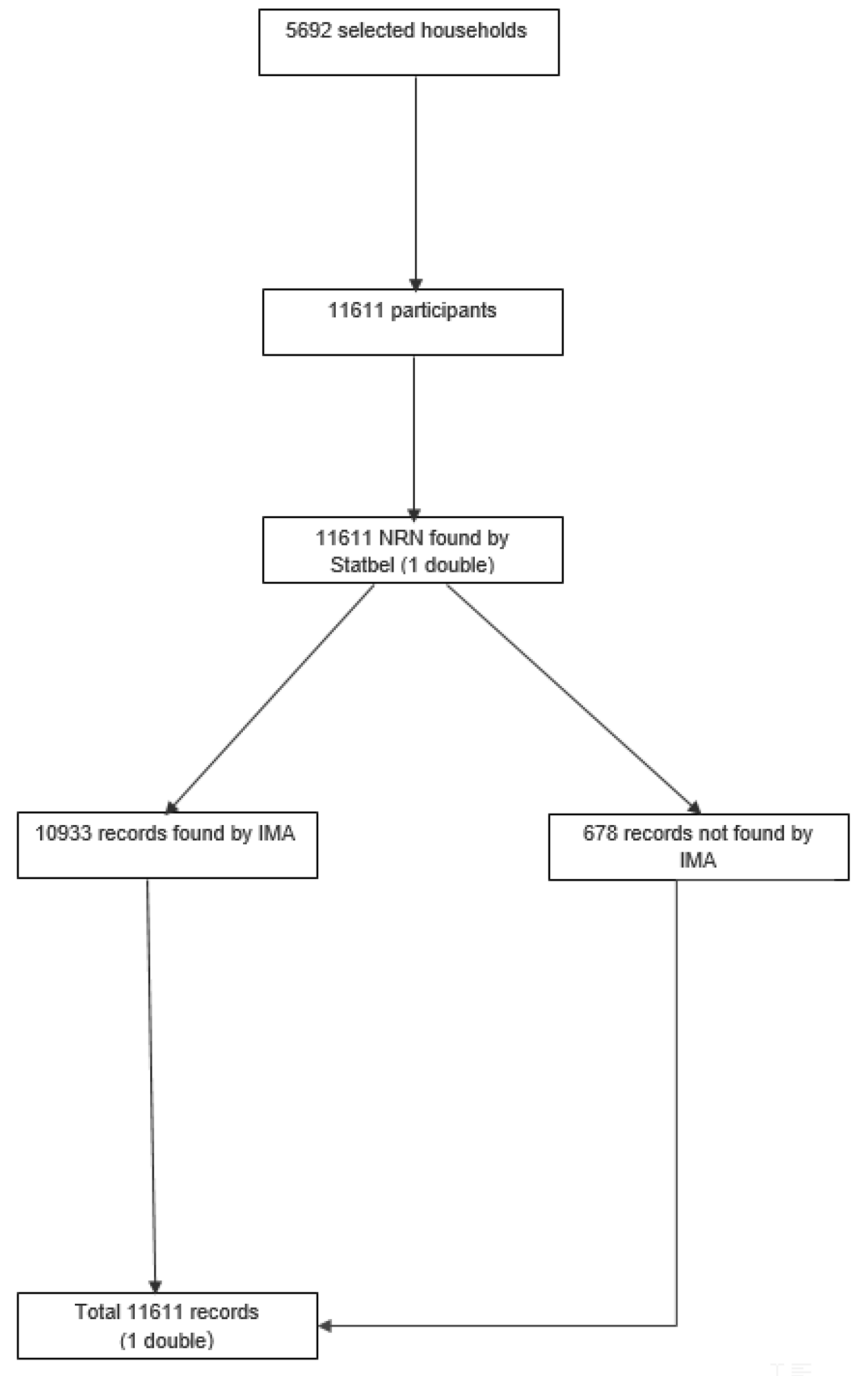
Data flow and linkage global results, HISlink 2018, Belgium *IMA: InterMutualist Agency, NRN: National Register Number*

Table 1 presents the linkage rates and the results of the evaluation of linkage quality. The linkage rates differed between population subgroups.

**Table 1:** Characteristics of the study population with linked and unlinked data, HISlink 2013 and 2018, Belgium

	HISlink 2013				HISlink 2018			
	Linked N = 9998	Unlinked N = 831	Standardized difference	Linkage rate (%)	Linked N = 10,933	Unlinked N = 678	Standardized difference	Linkage rate (%)
**Characteristics**								
***Gender, n (%)***								
Male	4819 (48.8)	412 (47.4)	0.03	92.1	5235 (49.0)	353 (56.7)	-0.16	93.7
Female	5179 (51.2)	419 (52.6)	-0.03	92.5	5698 (51.0)	325 (43.3)	0.16	94.6
***Age, n (%)***								
0–14	1523 (17.2)	193 (27.0)	-0.24	88.7	1766 (17.7)	92 (13.6)	0.11	95.0
15–24	1051 (11.5)	100 (13.1)	-0.05	91.3	994 (11.3)	65 (11.1)	0.01	93.8
25–34	1272 (12.3)	134 (15.3)	-0.08	90.5	1254 (12.8)	84 (15.7)	-0.08	93.7
35–44	1378 (13.7)	144 (14.8)	-0.03	90.5	1461 (12.7)	117 (12.9)	-0.01	92.6
45–54	1445 (14.9)	113 (13.3)	0.05	92.7	1569 (13.8)	156 (21.2)	-0.19	90.9
55–64	1379 (12.5)	71 (7.5)	0.17	95.1	1584 (13.1)	86 (14.8)	-0.05	94.8
65–74	998 (9.0)	34 (3.7)	0.21	96.7	1249 (9.7)	40 (5.4)	0.16	96.9
75+	952 (8.9)	42 (5.3)	0.14	95.8	1056 (8.9)	38 (5.3)	0.14	96.5
***Education, n (%)***								
Primary/No diploma	1054 (9.4)	79 (9.1)	0.01	93.0	779 (5.8)	32 (5.1)	0.03	96.0
Lower secondary	1389 (12.3)	64 (8.9)	0.11	95.6	1391 (12.2)	43 (6.9)	0.18	97.0
Upper secondary	3194 (33.3)	201 (24.6)	0.19	94.1	3279 (32.0)	123 (18.9)	0.30	96.4
Higher education	4211 (43.8)	468 (55.7)	-0.24	90.0	5309 (48.8)	446 (67.3)	-0.38	92.2
Missing	150 (1.2)	19 (1.7)	-0.05	88.7	175 (1.2)	34 (1.8)	-0.04	83.7
***Household composition, n (%)***								
Single	1685 (15.1)	78 (10.9)	0.12	95.6	2047 (15.5)	104 (14.1)	0.04	95.2
One parent with child(ren)	1115 (9.0)	87 (7.8)	0.04	92.8	1228 (10.9)	48 (6.6)	0.15	96.2
Couple without child(ren)	2203 (22.1)	125 (17.6)	0.11	94.6	2469 (22.4)	129 (24.2)	-0.04	95.0
Couple with child(ren)	4105 (45.2)	374 (45.9)	-0.01	91.6	4656 (46.3)	361 (51.5)	-0.11	92.8
Other or unknown	890 (8.6)	167 (17.8)	-0.27	84.2	533 (4.9)	36 (3.6)	0.07	93.7
**Nationality**, ***n (%)***								
Belgian	8834 (91.4)	457 (60.0)	0.79	95.1	9461 (90.1)	300 (50.1)	0.97	96.9
Non Belgian - EU	700 (4.9)	276 (29.1)	-0.68	71.7	846 (5.2)	338 (43.0)	-0.98	71.4
Non-Belgian - non EU	457 (3.6)	98 (10.9)	-0.28	82.3	621 (4.7)	40 (6.9)	-0.09	93.9
Missing	7 (0.1)	0 (-)	-	100.	5 (0.1)	0 (-)	-	100
**Household income**, ***n (%)***								
Quintile 1	1983 (16.9)	141 (21.3)	-0.11	93.4	1192 (8.9)	29 (6.0)	0.11	97.6
Quintile 2	1516 (14.9)	57 (10.9)	0.12	96.4	1450 (11.9)	26 (3.2)	0.33	98.2
Quintile 3	1748 (18.7)	93 (13.4)	0.14	94.9	1820 (16.5)	41 (8.3)	0.25	97.8
Quintile 4	1768 (20.5)	83 (12.8)	0.21	95.5	2322 (22.4)	84 (11.8)	0.28	96.5
Quintile 5	1781 (19.7)	193 (22.1)	-0.06	90.2	2487 (26.4)	317 (47.5)	-0.45	88.7
Missing	1202 (9.3)	264 (19.4)	-0.29	82.0	1662 (13.9)	181 (23.2)	-0.24	90.2
***Region of residence, n (%)***								
Flanders	3425 (57.9)	87 (32.0)	0.54	97.5	4230 (56.5)	66 (31.4)	0.52	98.5
Brussels	2715 (10.2)	388 (29.6)	-0.50	87.5	2873 (10.2)	226 (26.2)	-0.42	92.7
Wallonia	3858 (31.9)	356 (38.4)	-0.14	91.5	3830 (33.3)	386 (42.4)	-0.19	90.8

To assess the linkage quality, a comparison was made between the characteristics of linked and unlinked data [17, 35]. Standardized differences of the proportions were used to test for statistically-meaningful differences between those with linked and those with unlinked data [36–38]. The standardized difference was the difference in the two proportions, divided by an estimate of the prevalence of the covariate in each of the two groups [37]. A value equal to or greater than 0.10 was considered significant [37, 38]. Significant differences were observed between respondents with linked and unlinked records in terms of age, educational attainment, household composition, nationality, household income and the region of residence both for HISlink 2013 and HISlink 2018, while significant differences were observed according to gender, with a lower linkage rate for males, for HISlink 2018 only (Table 1).

## Outcomes of linked data - added values of HISlink for epidemiological research

Linking BHIS to BCHI data has resulted in a richer database, which has allowed studies to be carried out that would not have been possible using the two sources separately. Table 2 gives some examples of studies undertaken using the linked database. These examples illustrate the added value of the HISlink data for public health research. The studies carried out can be grouped in terms of different benefits or objectives in validation studies, policy-driven research studies and longitudinal studies.

**Table 2:** Examples of epidemiologic research with HISlink data, Belgium

Study	Research questions	Key findings	Reference
Validity of mammography uptake in women aged 50–69 years	To which extent self-reported mammography uptake from BHIS is valid as compared to objective information from IMA?	The validity of self-reported mammography uptake in women aged 50–69 years is affected by both selection and reporting bias. Cautiousness is needed when using self-reported estimates as the sole method to quantify mammography coverage.	Berete et al. (2020) [22]
Ascertainment of chronic diseases	Can the indicators of pseudopathologies in administrative data (IMA data) be used to assess prevalence of chronic diseases in the general population?	The indicators of pseudopathologies are an acceptable alternative to identify cases of diabetes, CVDs, Parkinson’s disease and thyroid disorders but yield in a significant underestimated number of patients suffering from asthma and COPD. Further research is needed to refine the definitions of CDs from administrative data.	Berete et al. (2020) [27]
Impact of financial protective measures on dental health care use	What is the effectiveness of financial protective measures on the use of dental care among a representative sample of Belgian adults?	Current health interventions are not yet effective for vulnerable people in dental care use. High expenses as a result of chronic diseases are not associated with more postponement of dental care. More targeted financial interventions should be necessary to reduce postponement of dental service utilization.	Berete et al. (2020) [39]
Nursing home admission in older population in Belgium	What is the risk of nursing home admission among older population of 65 + years in Belgium? What are the predictors?	The cumulative risk of NHA was 1.4%, 5.7% and 13.1% at, respectively 1 year, 3 years and 5 years of follow-up Higher age, living arrangements, use of home care services, falls, urinary incontinence, subjective health, limitations, depression, Alzheimer disease, etc., appeared as strong predictors nursing home admission.	(Berete et al., 2022) [29]
Mediation effects of health literacy	Does health literacy mediate the relationship between socioeconomic status and health related outcomes in the Belgian adult population?	HL partially mediated the relationship between education and health behaviour (except tobacco consumption), perceived health status, purchase of antidepressants and preventive dental care, accounting for 4.4–15.4% of the total effect. Health literacy also mediated the association between income health behaviour (except alcohol consumption), perceived health status, purchase of antidepressants and preventive dental care, with the mediation effects accounting for 4.2–12.0% of the total effect.	Berete et al. Will be submitted to BMC Public Health [40]
Assessing prevalence of polypharmacy among older adults	What is the differences in the prevalence and determinants of polypharmacy in the older population between self-reported and prescription based estimates? What is the relative merits of each data source?	Surveys and prescription data measure polypharmacy from a different perspective, but overall conclusions in terms of prevalence and determinants of polypharmacy do not differ substantially by data source.	Van der Heyden et al. (2021) [30]
Association between polypharmacy and mortality in older population	What is the association between polypharmacy and mortality in the community dwelling population of 65 + years in Belgium?	Polypharmacy affects the mortality of older people in relatively good health. A critical evaluation of polypharmacy in older people below 80 years and in people without severe functional limitations may reduce mortality in these population groups.	Van der Heyden et al. (2021) [42]
Costs associated with excess weight in Belgium	What are the annual health care and lost productivity costs associated with excess weight among the adult population in Belgium, using national health data?	BMI has a substantial societal economic burden in Belgium. Every year at least €4.5 billion are spent to cover the direct and indirect costs related to overweight and obesity. Policies and interventions are urgently needed to reduce the prevalence of overweight and obesity thereby decreasing these substantial costs.	Gorasso at al. [41]

### Validation studies

The linked data offered opportunities to answer methodological questions on the validation of survey information, such as the validity of self-reporting or conversely on the validation of administrative information. For instance, data on the mammography uptake is usually based on self-reports in population-based surveys such as BHIS. However, the validity of self-reported information through surveys is a concern, due to the associated potential reporting bias. To gain further insights into the validity of self-reported breast cancer screening in Belgium, we assessed the selection and reporting biases of BHIS-based estimates in the target group (women aged 50–69 years) using reimbursement data for mammograms taken from the BCHI. We found that the validity of self-reported mammogram uptake in women aged 50–69 years, is affected by both a selection and reporting bias (overreporting) and caution should therefore be exercised when using BHIS information as the sole source for assessing mammogram uptake [22].

Currently, the estimation of the prevalence of many chronic diseases in Belgium is still often based on self-reported BHIS data. On the NIHDI’s initiative, we evaluated whether BCHI data can be used to ascertain the prevalence of chronic diseases in the Belgian population. For this purpose, we assessed the agreement between the definitions used in health-administration cases (algorithms based on Anatomical Therapeutic Chemical (ATC) codes of disease-specific medication) and the definitions used in self-reported cases (based on the response to the following question: *“Have you suffered from any of the following diseases in the last 12 months?:“* diabetes, asthma, chronic obstructive pulmonary disease (COPD), cardiovascular diseases including hypertension (CVDs), Parkinson’s disease, thyroid disorders and epilepsy in the Belgian population. We concluded that BCHI’s chronic-disease case definitions are an acceptable alternative for identifying cases of diabetes, CVDs (including hypertension), Parkinson’s disease and thyroid disorders, but yield a significantly-underestimated number of patients suffering from asthma and COPD [27].

Another study explored the differences between self-reported and prescription-based estimates in the prevalence and determinants of polypharmacy in the older, general population in Belgium; and assessed the relative merits of each data source. The key findings were that surveys and prescription data measures polypharmacy from a different perspective, but overall conclusions in terms of prevalence and determinants of polypharmacy do not differ substantially according to data source [30].

### Policy-driven research

The linked database served as an evaluation tool for policy measures. Indeed, in our study *“*Effectiveness of protective measures on dental care use: analysis from linked database” we assessed the effectiveness of financial protective measures on the use of dental care among a representative sample of Belgian adults. We concluded that the current health interventions in dental care use are not yet effective for vulnerable people [39].

The reduction of socioeconomic (SE) health inequalities is an important objective for public health policies. It is therefore important to identify factors that contribute to these inequalities. Health literacy (HL) is of interest as it constitutes a potential pathway by which socioeconomic status (SES) affects health. In contrast to a number of socioeconomic factors that are more difficult to modify, HL is a more easily modifiable factor. As such, HL can also be taken into account in the attempt to reduce health inequalities. If HL is an important mediator in explaining SE health differences, actions to improve HL in low SE groups will reduce SE inequalities. This study explored whether HL acts as a mediator in the association between SES as measured by educational attainment and household income and a selected health (-related) outcomes that were of great interest from public health perspective in various domains: [1] health behaviour (physical activity, type of diet, alcohol and tobacco consumption) [2], perceived health status (self-rated health (SRH)) [3], use of curative care (purchase of antibiotics and antidepressants), and [4] use of preventive care (preventive dental care, influenza vaccination, breast cancer screening). The study showed that HL partially mediated the relationship between education and health behaviour (except tobacco consumption), perceived health status, purchase of antidepressants and preventive dental care, accounting for 4.4–15.4% of the total effect. As far as the association between household income and health (-related) outcomes is concerned, the findings showed that HL constituted a pathway by which household income influences health behaviour (except alcohol consumption), perceived health status, purchase of antidepressants) and preventive dental care, with the mediation effects accounting for 4.2–12.0% of the total effect [40].

The linked data has been used to estimate the annual costs in health care and lost productivity associated with excess weight among the adult population in Belgium. The study concluded that BMI is a substantial social-economic burden in Belgium. Every year at least €4.5 billion are spent to cover the direct and indirect costs related to excess weight and obesity. Policies and interventions are urgently needed to reduce the prevalence of excess weight and obesity, thereby decreasing these substantial costs [41].

### Longitudinal study

The linked data not only increases the number of variables. By following-up on BHIS participants up to 5 years after the survey, research questions can be addressed that require a longitudinal design. In this context, we estimated the risk of nursing home admission (NHA) among the older population of 65 + years and its predictors in Belgium. We found that the cumulative risk of NHA was 1.4%, 5.7% and 13.1% at, respectively 1 year, 3 years and 5 years of follow-up. A higher age, living arrangements, falls, physical chronic conditions and mental disorders such as Alzheimer’s disease, appeared as strong predictors of NHA [29].

The HISlink data was further used to investigate the association between polypharmacy and mortality in the community-dwelling older population. It was found that polypharmacy affects the mortality of older people who are in relatively good health and concluded that a critical evaluation of polypharmacy in older people aged below 80 years and in people without severe functional limitations may reduce mortality in these population groups [42].

## Lessons learned and recommendations for future linkages

Although linking survey data with administrative data opens new research opportunities as presented above, such linkage is not without challenges. This section describes the main challenges and considerations that may be encountered in data linkage processes and a number of recommendations for future linkages will be formulated. Table 3 provides a summary of the challenges and considerations and the corresponding recommendations.

**Table 3:** Table Overview of challenges, considerations and recommendations in linking surveys data with administrative data; HISlink, Belgium

Category	Description	HISlink-specific experience	Recommendations
**Technical, practical challenges**			
Data quality	Availability, completeness and discriminatory power of identifiers	National register number available and used as linkage key	Use unique identifier when available. Otherwise, carefully select linkage variables to construct linkage the keys. Ensure that these variables are as complete as possible (less missing values, less errors) and that no duplicate records exist in each data source.
Linkage errors	Usually arises in data linkage, typically when ‘imperfect identifiers’ are used and could result in substantially biased results. False matches (i.e., when records from different individuals link erroneously) and missed matches (i.e., when records from the same individual fail to link) [45, 46] are of greatest concern. The number of false matches and missed matches can directly affect the estimation of prevalence or incidence rates. False matches (low specificity) lead to overestimates of prevalence whilst missed matches (low sensitivity) lead to underestimates. The impact of linkage error depends on the underlying prevalence of the target condition: analyses of rare conditions are more severely affected by linkage error compared with more common conditions, as overestimation is inversely related to the underlying prevalence [46].	Negligible/marginal false matches because of the accuracy of the linkage key. However, up to 8% of missed matches (see section 4.1 for possible explanations). The comparison of linked and unlinked records identified subgroups that are more prone to linkage errors (see Table 1).	Evaluate linkage quality and assess the impact of linkage errors on the results [17, 35, 46]. The evaluation of linkage quality is vital to producing reliable results from studies using the linked data. Several methods can be used to assess linkage quality and errors: - comparing linked data with reference or ‘gold-standard’ datasets where the true match status is known; - structured sensitivity analyses where a number of linked datasets are produced using different linkage criteria; - comparisons of characteristics of linked and unlinked data to identify any potential sources of bias; - statistical methods accounting for linkage uncertainty within analysis (e.g. using missing data methods); - quality control checks (implausible scenarios) - sensitivity (proportion of matches that are correctly identified as links), specificity (proportion of non matches that are correctly identified as non-links), match rate and false match rate. The TTPs should enhance the linkage methods by combining deterministic linkage in the first steps using the NRN and probabilistic approaches afterwards for unlinked persons using algorithm based on other personal data. Identify subgroups of records that are more prone to linkage error and are potential sources of bias. Comparisons of linked and unlinked records can be useful to identifying where modified linkage strategies may be required for specific groups of records. Use the NRN of all individuals included in the survey, regardless of the composition of the household at one time, instead of that of the reference person first and then the other family members, in order to improve the linkage rate.
Costs	Data linkage can be expensive in terms of financial and human resources.	Government-sponsored (NIHDI) linked datasets	Make the system cost-effective by avoiding the ‘linked and destroyed’ philosophy and making available the linked data to other researchers under certain conditions.
Principle of proportionality respect	Means that only data that are relevant to the purpose of the study should be included to avoid re-identification of individuals.	Help from the BHIS team for the selection of BHIS variables and help from the IMA’s SPOC for what concern IMA variables.	Require a deep knowledge of the data sources. Involve people with good experience of the data sources to be linked in the relevant variable selection phase. An alternative and more effective approach could be could be too ask for authorization to link both datasets completely in a first step. In a second step, each research project demands in a simplified procedure access to the relevant variables of the fully linked dataset in accordance with the proportionality principle. Such an approach is applied at Statistics Netherlands (49–51).
Infrastructures	Infrastructure needed to store and access the linked data.	The linked data was stored on the IMA server. Researchers access it through a secure remote connection using a token.	Identify where linked data can be stored securely and how it can be accessed (remote session, data extraction).
Statistical issues	Analysing linked data raises a number of statistical challenges for researchers.	Experts’ advice during the statistical analysis plan, data analysis and interpretation of results.	Experts’ advice useful for the statistical analysis plan, data analysis and results interpretation. Apply appropriate statistical methods of adjusting analysis for linkage bias. E.g., an extension to standard multiple imputation methods, able to handle ‘partially observed’ (or partially linked) data; use of population weights to account for groups or people who are more or less likely to be linked [46].
**Ethical, legal and societal aspects**			
Approval processes	Privacy concerns have led to policies that prevent records from being easily linked. Usually, there is a need of intuitional/ethical review boards (IRB) approval which is a long and cumbersome process.	The linkage was approved by the Information Security committee (ISC). The approval process took three and five months for the HISlink 2013 and HISlink 2018, respectively.	Consider the IRB process in the timeline for the project. Concerns about privacy led to policies that prevent records from being easily linked. Therefore, a strong case for using the data and a detailed description of how it will be protected is required when obtaining IRB approval. Since the HISlink is government-sponsored linkage project which is repeated every BHIS wave, a solution to avoid an ad hoc approval process would be to set up an “umbrella” agreement protocol for public institutions such as Sciensano, covering several years and several waves of BHIS_BCHI linkages.
Privacy and confidentiality issues: actual linkage process and principle of separation (Trusted Third Party linkage)	Once the IRB approval has been obtained, the actual linkage is itself a time-consuming process. The separation principle means a separation of the linking and analysis process. Although this principle preserves confidentiality and avoids disclosing sensitive information, it is bad for understanding the quality of linked data.	Trusted Third Party linkage, a lengthy process mainly due to the signing of an agreement between all parties involved. The whole linkage procedure took 12 months and 15 months for HISlink 2013 and HISlink 2018, respectively.	Although full separation of identifiers and attribute data has been argued to reduce the risk of re-identification, and is a valuable tool in reassuring data providers about the security of sharing their data. However, allowing linkage and analysis to take place together provides opportunities for both in-depth evaluation of linkage quality, and methodological advances in linkage technics [76, 77].
Consent form	To comply with the GDPR, an effective opt-in linkage consent form have to be received.	HISlink 2013 and 2018 were not consent-based (exemptions, linkage planed before the implementation of the GDPR). However, for the next HISlink 2023, the consent of the BHIS participants was asked to link their data with existing administrative data.	For planned linkage, ask for linkage consent to the survey participants, preferably at the beginning of the survey to maximise consent rate [55, 68, 69]. For historical data linkage, certain exemptions exist. Check if the project falls under these exemptions. Assess consent bias if applicable
**Outcomes**			
Opportunities / limitations of linked data	The linked data is an important source for population health research and can bring enormous benefits in providing a more complete picture of the health of the population. A whole range of research possibilities exists.	Limitations of both BHIS and BCHI data remain, for instance lack of diagnostic information in the BCHI data	Include other data sources such as hospital discharge data Consider substituting HIS information by administrative data as much as appropriate (e.g., or cancer screening, reimbursed, healthcare use or reimbursed drug use).
Linkage type and sustainability	Ad hoc linkages vs. systematic linkages	Ad hoc linkage (and ad hoc approval) can threat the sustainability of the project. HISlink is based on the ‘linked and destroyed philosophy’ (because of a limited data retention time by researchers in the IRB approval, i.e., five years after the linkage) As a result, the return on investment in linked data may be limited.	A clear data use agreements for governmental institutions, administrations, universities allowing share and use of the linked databases for at least several years even if for perpetuity in a secure manner. Such strategies will allow to exploit the full potential of the linked data in other researches. Think about systematic linkage.
Access to the linked data		HISlink data is currently accessible to Sciensano researchers only.	Make de-identified data available to other researchers upon approval
Sample size	Small sample can prevent some analyses	Limited sample size for rare events, specific subgroup analysis	Consider subsample for specific subgroups such as low sociodemographic individuals, those with specific conditions if possible.

### Lessons learned from to the linkage processes overall

#### Technical and operational issues of the linkage

The technical challenges inherent in linking survey data with administrative data are mainly related to the data quality and to the linkage errors [43]. Next to these issues, the proportionality principle, infrastructure and statistical challenges are also important.

The quality of the data sources, i.e., the availability, completeness and discriminatory power of identifiers or key personal variables that can be used to construct the linkage key, is very important and determines the choice of linkage methods.

In some countries, a unique personal number, such as the NRN in Belgium or the personal identity number in Scandinavia, is required for access to almost all administrative services, including healthcare services use for each resident and can be readily used to obtain information about individuals. Such identifiers allow the linkage to be relatively straightforward (deterministic linkage approach), and make it possible to link data from many different administrative sources with marginal error [44]. With regards to HISlink, the use of the NRN as a linkage key was a great asset. Moreover, such a unique identifier increases the linkage rate, although this rate varies between subgroups as shown in Table 1. About 8% of the BHIS2013 and 6% of the BHIS2018 could not be linked. This result could be explained by the fact that the BHIS household composition can deviate from the “official” household composition in the national register, preventing the linkage. In addition, as Table 1 shows, the linkage was not possible for a number of people who are more likely to be from the Brussels-Capital Region and more likely to be EU nationals. This sub-group could probably be people working for EU institutions, other international organizations or posted workers from other EU countries, living and working in Belgium but insured in their country of origin. Therefore no data could be retrieved from the BCHI.

In many other countries however, unique identifiers are not available and this might constitute an important barrier to linking the same person across multiple data sources [18]. In such contexts, linkage often depends on the use of non-unique ‘imperfect’ identifiers such as name, postcode, date of birth or other indirect identifiers. In combination, these variables can make it possible to identify records that belong to the same person, using more complex algorithms (probabilistic linkage approach). The probabilistic linkage method is the most common approach, usually in combination with the deterministic methods [45, 46].

The second challenge when linking survey data to administrative data is the risk of linkage errors, which typically occur where there is no unique identifier across different data sources [47] or in the event of imperfect identifiers. This problem could result in substantially biased results [17, 48]. Linkage errors arise when pairs of records are incorrectly classified. False-matches occur when records from different individuals link erroneously, while missed-matches occur when records from the same individual fail to link [45, 46]. Data analysts should therefore evaluate the quality of linked data by measuring linkage errors before proceeding with any further analysis. The availability of similar information in both data sources or in a reference database will be helpful in this regard. For HISlink, comparing age, sex, region of residence and the prevalence of certain chronic diseases, we detected an error in the previous version of HISlink 2018 data due to the use of the wrong database during the linkage process. This error was corrected by the linkage TTPs afterwards.

Another challenge that researchers face in data linkage is the proportionality principle, which means that only those variables that are relevant to the purpose of the study should be selected to avoid the re-identification of individuals. In this context, researchers should have a thorough knowledge of their data sources. The selection of relevant variables must be done precisely before the linkage process. The more information there is in both data sources, the more difficult this task becomes. However, this approach is not optimal as it is time-consuming and requires an in-depth knowledge of the data sources. In addition, when it is necessary to include new relevant variables or indicators that have been forgotten, the whole process has to be restarted (new IRB opinion, new linkage, etc.). An alternative, perhaps better approach could be too ask for permission to link both datasets completely in a first step. In a second step, each research project demands in a simplified procedure access to the relevant variables of the fully linked dataset in accordance with the proportionality principle. This is basically what is done at Statistics Netherlands [49–51].

Further consideration for researchers wishing to link data is the infrastructure needed to store and access the linked data. Some questions need to be answered beforehand: how will data be stored safely? What is the cost for the infrastructure? How will data be protected? How can data be accessed in a safe and easy way [28]?. In the case of HISlink, the linked data was stored on the IMA server and researchers access it securely using a token.

Finally, analysing linked datasets raises a number of additional ‘statistical’ challenges for researchers. Although linked data has several advantages, it is important to bear in mind that the limitations of both data sources remain even after the linkage. Researchers need to be aware of this to understand and interpret the results carefully. In addition, in the event of linkage errors, specific statistical methods need to be applied [35, 46]. Furthermore, with the complexity of administrative data, it is often necessary to involve an expert on this data in the analysis stages as well as when interpreting the results. In our case, the BCHI data is collected for administrative purposes, not for epidemiological research. It is therefore not easy to understand and use. Expert advice is often needed to make good choices when planning the analysis. The IMA’s single point of contact and the many experienced Sciensano researchers are well-qualified to fulfil this requirement.

#### Ethical, legal and societal aspects

The most important concerns facing data linkage are privacy and confidentiality issues [52]. With the implementation of the GDPR in 2018, new decision-making bodies were established for the authorisation of data linkage, and privacy and confidentiality issues were redefined. Because of these confidentiality issues, institutional review board (IRB) approval is often required to link the data. However, such IRB approval processes are usually complex and time-consuming, especially when the linkage is not consent-based. For both HISlink 2013 and HISlink 2018, it took several months to get the IRB approval. Therefore, to facilitate data linkage and overcome the lengthy negotiation and ad hoc approval processes for each BHIS-BCHI linkage, it would be useful to set up some kind of umbrella agreement protocol for public institutions such as Sciensano, to cover several years and several waves of BHIS-BCHI linkages.

To preserve privacy and prevent the disclosure of sensitive information, data linkage often relies on the separation principle of linkage and analysis processes, meaning that those conducting the linkage (often TTPs) only have access to a set of identifiers, whilst those analysing the linked data only have access to de-identified attribute data [17]. However, this type of approach causes a significant delay in the linkage process due to the administrative steps that take time (e.g. the signature of an official agreement between the parties involved). Furthermore, although this approach reduces the risk of disclosure of sensitive information about individuals, it means that important aspects of the linkage process are obscured, which makes it difficult for researchers to judge the reliability of the resulting linked data for their required purposes [17, 47].

Respecting respondents’ rights and maintaining their trust are further considerations. According to the new EU data Act, trust and altruism are essential in secondary data use [53]. When researchers plan to link data as part of a future survey, citizens must be able to decide whether they want to share their data, they must be informed that their data is being used and by whom. In other words, they need to opt-in through informed consent [1, 9, 54, 55]. Informed consent is required to ensure that respondents are aware of the risks and benefits involved in releasing and linking their personal data for research purposes, even though obtaining the opt-in linkage consent from all respondents is a challenging task. To link historical survey data to administrative data, there are exceptions to the requirement for informed consent, especially if contacting study participants is impossible or unreasonable [1, 9]. The GDPR contains specific exemptions to informed consent as a legal basis for the use of data to escape a ‘consent or anonymise approach’ or a ‘fetishisation of consent’, especially in the case of observational health research [56]. For the BHIS2013 and BHIS2018 linkages, because of the disproportionality to inform and seek consent from all BHIS participants and also because the authorization procedure was implemented prior to the GDPR, we proposed that the acquisition of consent from BHIS participants was obtained by way of a waiver, and this approach was accepted by the IRB. While these exemptions to informed consent are possible for historical data linkages, for any planned future linkages, researchers must seek informed consent from participants during the survey.

### Lessons learned related to the outcomes

Without a doubt, the HISlink offers the potential to obtain more comprehensive data on the population’s health, facilitating new research perspectives for public health as demonstrated in this study. The BHIS data are only available every 5 years and some studies require more comprehensive data than the current linked data. The HISlink can be seen as a first step towards more comprehensive data linkages. To ensure that the benefits of data linkage are fully maximised, it is important to consider the inclusion of other administrative data such as hospital discharge data, mortality data, environmental data, primary electronic medical record (EMR), etc. For example, extending linked data to hospital discharge data could help target internal quality improvement efforts for specific patient groups (e.g., preventive care for diabetics) or help assess the determinants of hospitalisation and understand the underlying factors that influence length of hospitalisation. A linkage with the EMR may also be useful for studying appropriate polypharmacy, for example. However, in some countries such as Belgium, there is currently no integrated primary EMR. Only a few sentinel networks exist, such as the Intego database. For the future, consideration needs to be given to establishing a legal framework for such an integrated database.

At international level, the linkage between survey and administrative data has also proven its value. Indeed, such a linkage has been widely used in validation studies [10, 57, 58], but also in addressing specific research questions. For example, using health survey data linked to administrative health services data, the Institute for Clinical and Evaluative Sciences (ICES) researchers in Ontario, Canada, developed and validated an algorithm for population-based prediction of diabetes - the Diabetes Population Risk Tool (DPoRT) that accurately predicts diabetes risk in a population [59]. The linkage of Canadian Community Health Survey (CCHS) with medical claim data, has been used to investigate individual-level characteristics that are associated with community-dwelling high-cost users. They found that high-cost users status was strongly associated with being older, having multiple chronic conditions, and reporting poorer self-perceived health. The authors further found that high-cost users tended to be of lower socio-economic status, former daily smokers, physically inactive, current non-drinkers, and obese [60]. Finally, the linkage of survey and administrative data has been used to address methodological issues such as bias adjustment [61–63] or non-response analysis [64].

The BCHI data does not contain clinical information. In addition, there is no information on non-reimbursed care in the BCHI data. Although information is available on vital status, there is no information on cause of death. The absence of such important information prevents some policy-oriented research questions from being answered better. In future, efforts could be made to include more data sources in HISlink, and an initial step would be to include hospital discharge data.

The BCHI data is only available two years after consumption, meaning that the linkage can only be made with a two-year delay which precludes ‘real time’ linkage. Data availability should be accelerated in the short to medium term given the widespread use of electronic billing.

Furthermore, with the limited sample size of the BHIS (about 10,000 participants), subgroup analysis is impossible or yields inaccurate results, for example for rare events or specific subgroups.

Finally, access to linked data is thus far highly restricted due to legal constraints. Only Sciensano researchers that are registered with the IMA as the users of the linked data have access to the data. To take further advantage of the linked data, the data owners, i.e., Sciensano, the IMA and the sponsor (NIHDI) could retain ownership but make the data available to other research studies in line with the primary objective of HISlink, subject to the owners’ approval. One example of such an approach in cancer research is the National Cancer Institute’s (NCI’s) linked Surveillance, Epidemiology and End Results (SEER)-Medicare files where the NCI retains ownership of the data and releases it for approved research studies that guarantee the confidentiality of the patients and providers in the SEER areas [65].

### Recommendations for future linkages

This study provides important information with regard to the individual linkage of survey data and health-insurance administrative data that other studies can build on. Based on our experience, there are a number of aspects that need to be taken into account to ensure the success of data linkage in future research. The recommendations related to the ethical, legal and societal aspects, technical, practical challenges, as well as those related to the outcomes are summarized in Table 3, and the main ones are further elaborated below.

#### Recommendation 1: gain and maintain the citizens trust in secondary use of data and data linkage

With the implementation of the GDPR, the consent form became mandatory for future planned linkages. Researchers need to put in place strategies to gain the trust of and to involve citizens whose data will be linked [66]. The perceived risk to privacy and data confidentiality constitutes one of the primary reasons why respondents decline the linkage request [55]. It is therefore important to emphasise the merits of the research, to stress the importance of altruism (contribution to society) and to address respondents’ privacy and confidentiality concerns by informing them of the safeguards put in place to protect their data.

#### Recommendation 2: improve the communication with the participant, so there is more willingness to give a consent for linkage

The literature suggests a strong correlation between respondents’ understanding and how likely they are to give consent [55, 67]. To achieve higher consent rates, it is necessary to shed light on respondents’ understanding of the linkage consent. Several approaches have been proposed to improve linkage consent rates. One of these consists of providing key subgroups that are less likely to understand the linkage request, with additional targeted explanatory or informative material. Another approach would be to use tailored messages by asking the consent understanding questions first, then doing a targeted intervention to address any misunderstandings, before administering the linkage request. It is preferable to ask for linkage consent upfront, which yields higher consent rates [9, 45, 50, 51].

#### Recommendation 3: adapt the need for consent to the context of the linkages

For linkages between datasets that already exist, a clear framework of acceptable practices needs to be developed, which the European Health Dataspace initiative is attempting to do [70]. To maintain population trust in secondary use of data and data linkage, it is imperative that this framework is in line with citizens’ values [66]. A clear distinction should be made between:

1) Routine linkages, which are usually for primary use and where implicit consent can be assumed because it concerns direct clinical care. However, a harmonized framework needs to be developed in order to streamline secure data flows;

2) Necessary linkages, in a public health crisis, as exemplified by the COVID-19 pandemic and where consent should not be required [71]; and.

3) Linkages for public health research and surveillance or other scientific research in the public interest, where the preferred legal basis should not be consent, but an explicit legal and ethical framework that is developed by the national health data authorities, resulting in a federated network of Findable, Accessible, Interoperable and Reusable (FAIR), linkable data sources governed by rules that are trusted both by researchers and citizens.

#### Recommendation 4: advoid the ‘link and destroy model’

Many challenges remain before this can become a reality, but it would resolve the administrative burden, the need for case-by-case consideration and the overall uncertainty and inefficiency surrounding data linkage [72]. From a broader perspective, it will be useful to have streamlined approval processes for efficient data access. Indeed, some jurisdictions adopt approaches for timely and cost-effective access to linked data (e.g. those in Ontario, Wales and Australia where linkage keys can be held in perpetuity), others such as in Belgium are restricted by the ‘link and destroy’ model, where linked data cannot be reused or are destroyed after a predefined dataretention time. In turn, these impact on the availability and accessibility of data for research and policy development (17).

#### Recommendation 5: take up initiatives to work towards a better balance between the right to privacy of respondents and society’s right to evidence-based information to improve health

Privacy considerations must strike a balance between the privacy rights of respondents and society’s right to evidence-based information to improve health.

Although the separation principle of linkage and analysis processes (as implemented at: the Data Linkage Branch in Western Australia, the Centre for Health Record Linkage (CHeReL) in New South Wales [73], the Secure Anonymous Information Linkage (SAIL) Databank in Wales [74], the Centre for Data Linkage (CDL) in Australia [75], the Manitoba Centre for Health Policy in Canada [73]) is recognised as good practice for protecting confidentiality, allowing linkage and analysis to take place together provides opportunities for both in-depth evaluation of linkage quality, and methodological advances in linkage techniques [76, 77]. Such an approach is in operation at the Institute for Clinical Evaluative Sciences (ICES) in Ontario. The ICES is legally allowed to receive fully identifiable data in order to perform linkage, to assess data quality and to provide coded data to research staff within the organisation. They operate a hierarchical access policy, which means that only a specific number of people have the highest level of access to all data elements, and most researchers can only access de-identified, coded data relevant to their study [73]. The linkage approach as applied at Statistics Netherlands constitutes a good practice in Europe [49–51].

#### Recommendation 6: optimize the way to deal with ethical and privacy requirements in order to be able to carry out data linkages in a reasonable time

Beside the privacy and confidentiality issues, researchers should be aware of some technical aspects such as the complexity of the linkage process which often results with a delay in the linkage process. Getting the agreement signed between the parties involved was a crucial factor in delaying the process, especially when several parties are involved. Therefore, a formal, pre-established accreditation that negates the need for new signatures at each linkage (ad hoc approval) for institutions that are entitled to request a data linkage, would be a further step towards reducing the delay and facilitating the data linkage process.

#### Recommendation 7: plan ahead the linkage of survey and administrative data, particularly where there is no unique identifier that can be used as a linkage key

If the linkage cannot rely on a unique identifier, researchers should identify more relevant variables (e.g., age, gender, date of birth, name, etc.) that will allow the construction of an almost perfect identifier for probabilistic linkage. As data linkage often relies on the separation of linkage and analysis processes, researchers should assess the linkage errors and quality of the linked data before conducting any further analysis. Several methods can be used to evaluate linkage quality, including the use of gold standard or reference data, sensitivity analyses, a comparison of the characteristics of linked and unlinked data, or post-linkage data validation [17, 35].

#### Recommendation 8: apply strategies to improve the linkage rates

Although the use of deterministic linkage methods has resulted in a relatively higher linkage rate, this approach is known to give rise to a number of missed matches (e.g. in the case of even a single digit error in the NRN). Therefore, a combination with subsequent probabilistic methods for unlinked cases to the deterministic linkage step would certainly result in a higher linkage rate. In addition, another explanation why the linkage was not always possible for everyone would be that only the NRN of the reference person was available and the others had to be found on the basis of household composition and socio-demographic characteristics. This approach is probably linked to the BHIS sampling strategy. However, BHIS household composition may differ from BCHI household composition or may change over time. Therefore, including the NRN of all individuals included in the survey, regardless of household composition would probably improve the linkage.

#### Recommendation 9: demonstrate to funders and policy makers the usefulness of linkages, raise awareness of such initiatives and continue to promote the linkage between databases

The linked data is an important source for population health research. Its use by researchers can bring huge benefits in terms of providing a more complete picture of the population’s health. However, within the context of budgetary constraints, it is important for researchers to demonstrate to funders and policy makers the usefulness of such linkage in order to maintain project funding and sustainability and to raise awareness of such initiatives. From a public health perspective, policy makers should continue to invest in data linkages; and the inclusion of other data sources (such as primary-care data and hospital discharge data) will augment the use of the linked data to expand the evidence base for policy makers and practitioners, which could therefore enrich population-based surveillance and research in the field of public health. However, in that case, there is a need to develop an overarching infrastructure. Since making linkages between multiple datasets would be very challenging, to be really cost-effective, it would be better to have an infrastructure that would allow access to different research institutes.

#### Recommendation 10: consider substituting HIS information by administrative data as much as appropriate

In view of the current challenges facing surveys, there is need to keep survey questionnaires as short as possible. Hence the more information can be obtained through other sources, the shorter can be the questionnaire. When possible, self-reported items should be replaced by administrative data. This will be the case, for example, for cancer screening, reimbursed healthcare use or reimbursed drug use. However, it is important to keep in mind that the replacement of self-reported information by administrative data can have certain limitations since administrative data have their own shortcomings (e.g., incomplete or missing data, recording errors).

## Conclusions

Data linkage provides important added value for public health researchers. From a public health perspective, policy makers should continue investing in data linkages; and the inclusion of other data sources such as primary care data and hospital discharge data will augment the use of the linked data to expand the evidence base for policy makers and practitioners, and can thus enrich population-based surveillance and the field of research into public health. Considering the strengths and limitations of different data sources, the opportunity to link several data sources could potentially enable a wider range of research questions to be addressed. However, linking survey data to administrative data is not without its challenges and these have to be tackled. Although some aspects of the HISlink may be specific to the Belgian context, we believe that this study has a much broader application and could be useful to researchers who plan to link health survey data with health administrative data for their respective projects.

### Electronic supplementary material

Below is the link to the electronic supplementary material.

Supplementary Material 1

## Data Availability

The survey datasets and linked health administrative data analysed in the current study are not publicly available due to restrictions in the General Data Protection Regulation (GDPR) on sensitive data such as personal health data. BHIS data contains sensitive and identifying information and must therefore only be made available upon request. Requests for data access may be made to the Social Security and Health Chamber of the Information security committee (hereinafter referred to as the “Social Security and Health Chamber”). Further information regarding the survey and the data access procedure can be found here: Health Interview Survey | Microdata request procedure | sciensano.be.

## List of abbreviations

ATC: Anatomical Therapeutic Chemical
BCHI: Belgian Compulsory Health Insurance
BelHES: Belgian Health Examination Survey
BHIS: Belgian Health Interview Survey
CAPI: Computer-Assisted Personal Interview
COPD: Chronic Obstructive Pulmonary Disease
CVD: Cardiovascular Diseases
GDPR: General Data Protection Regulation
HISlink: A linkage between BHIS data and BCHI data
IMA: Inter Mutualist Agency
IRB: Institutional Review Board
KCE: Belgian Health Care Knowledge Centre
NHA: Nursing Home Admission
NIC: National Intermutualist College
NIHDI: National Institute for Health and Disability Insurance
SCRA: Small Cell Risk Analysis
TTP: Trusted Third Party

## References

[1] March S, Andrich S, Drepper J, Horenkamp-Sonntag D, Icks A, Ihle P (2020). Good Practice Data linkage (GPD): a translation of the German version. IJERPH.

[2] Druschke D, Arnold K, Heinrich L, Reichert J, Rüdiger M, Schmitt J (2020). Individual-level linkage of primary and secondary data from three sources for comprehensive analyses of low Birthweight effects. Gesundheitswesen.

[3] Centre for Health Record Linkage (CHeReL). New South Wales (NSW) Gouvernment Website - Centre for Health Record Linkage. [cited 2023 Feb 9]. How record linkage works. Available from: https://www.cherel.org.au/how-record-linkage-works#:~:text=How%20record%20linkage%20works,of%20health%20events%20for%20individuals.

[4] Brook EL, Rosman DL, Holman CDJ. Aust N Z J Public Health. 2008;32(1):19–23. Public good through data linkage: measuring research outputs from the Western Australian Data Linkage System. 10.1111/j.1753-6405.2008.00160.x 18290908

[5] Tew M, Dalziel KM, Petrie DJ, Clarke PM (2017). Growth of linked hospital data use in Australia: a systematic review. Aust Health Review.

[6] Young A, Flack F (2018). Recent trends in the use of linked data in Australia. Aust Health Review.

[7] Maret-Ouda J, Tao W, Wahlin K, Lagergren J (2017). Nordic registry-based cohort studies: possibilities and pitfalls when combining nordic registry data. Scand J Public Health.

[8] Haneef R, Delnord M, Vernay M, Bauchet E, Gaidelyte R, Van Oyen H (2020). Innovative use of data sources: a cross-sectional study of data linkage and artificial intelligence practices across European countries. Arch Public Health.

[9] March S. IJERPH. 2017;14(12):1543. Individual Data Linkage of Survey Data with Claims Data in Germany—An Overview Based on a Cohort Study. 10.3390/ijerph14121543 PMC5750961 29232834

[10] Hall HI, Van Den Eeden SK, Tolsma DD, Rardin K, Thompson T, Hughes Sinclair A (2004). Testing for prostate and Colorectal cancer: comparison of self-report and medical record audit. Prev Med.

[11] Van der Heyden J, Charafeddine R, De Bacquer D, Tafforeau J, Van Herck K (2016). Regional differences in the validity of self-reported use of health care in Belgium: selection versus reporting bias. BMC Med Res Methodol.

[12] Van der Heyden J, Van Oyen H, Berger N, De Bacquer D, Van Herck K (2015). Activity limitations predict health care expenditures in the general population in Belgium. BMC Public Health.

[13] Mimilidis Hélène D, Stefaan T, Jean. Van Der Heyden Johan. Projet De couplage de données issues de l’Enquête de Santé 2008 et des organismes assureurs. Bruxelles, Belgique;; 2014. Mai. Report No.: D/2014/2505/32.

[14] Holman CDJ, Bass AJ, Rouse IL, Hobbs MST (1999). Population-based linkage of health records in Western Australia: development of a health services research linked database. Aust N Z J Public Health.

[15] Holman CDJ, Bass JA, Rosman DL, Smith MB, Semmens JB, Glasson EJ (2008). A decade of data linkage in Western Australia: strategic design, applications and benefits of the WA data linkage system. Aust Health Review.

[16] Mirel LB. The NCHS Data Linkage Program: Leveraging the nation’s health data for evidence-based decision making. In 2020 [cited 2022 Jun 27]. p. 28. Available from: https://www.cdc.gov/nchs/data/datalinkage/Data-Linkage-Webinar.pdf

[17] Harron K, Dibben C, Boyd J, Hjern A, Azimaee M, Barreto ML (2017). Challenges in administrative data linkage for research. Big Data & Society.

[18] Harron K (2022). Data linkage in medical research. Bmjmed.

[19] Harron K, Gilbert R, Cromwell D, van der Meulen J. Linking Data for Mothers and Babies in De-Identified Electronic Health Data. Gebhardt S, editor. PLoS ONE. 2016;11(10):e0164667. 10.1371/journal.pone.0164667 PMC5072610 27764135

[20] Demarest S, Van der Heyden J, Charafeddine R, Drieskens S, Gisle L, Tafforeau J (2013). Methodological basics and evolution of the Belgian health interview survey 1997–2008. Arch Public Health.

[21] Van der Heyden J. Validity of the Assessment of Population Health and Use of Health Care in a National Health Interview Survey. [Ghent, Belgium]: Ghent University - Faculty of medicine and health sciences; 2017 [cited 2023 Feb 9]. Available from: https://biblio.ugent.be/publication/8523878

[22] Berete F, Van der Heyden J, Demarest S, Charafeddine R, Tafforeau J, Van Oyen H (2021). Validity of self-reported mammography uptake in the Belgian health interview survey: selection and reporting bias. Eur J Pub Health.

[23] Hunger M, Schwarzkopf L, Heier M, Peters A, Holle R, KORA Study Group (2013). Official statistics and claims data records indicate non-response and recall bias within survey-based estimates of health care utilization in the older population. BMC Health Serv Res.

[24] Devos C, Cordon A, Lefevre M, Obyn C, Renard F, Bouckaert N (2019). Performance of the Belgian health system–report 2019. Health Services Research (HSR).

[25] Noordhout CMD, Devos C, Adriaenssens J, Bouckaert N, Ricour C, Gerkens S. Health system performance assessment: care for people living with chronic conditions.

[26] Bouckaert N, Maertens de Noordhout C, Van de Voorde C. Health System Performance Assessment: how equitable is the Belgian health system?. Brussels: Belgian: Health Services Research (HSR). Health Care Knowledge Centre (KCE); 2020 [cited 2022 Jun 27] p. 105. Report No.: KCE Reports 334. D/2020/10.273/30. Available from: https://kce.fgov.be/sites/default/files/2021-11/KCE_334_Equity_Belgian_health_system_Report.pdf

[27] Berete F, Demarest S, Charafeddine R, Bruyère O, Van der Heyden J (2020). Comparing health insurance data and health interview survey data for ascertaining chronic Disease prevalence in Belgium. Arch Public Health.

[28] Maetens A, De Schreye R, Faes K, Houttekier D, Deliens L, Gielen B (2016). Using linked administrative and disease-specific databases to study end-of-life care on a population level. BMC Palliat Care.

[29] Berete F, Demarest S, Charafeddine R, Ridder K, Vanoverloop J, Oyen H et al. Predictors of Nursing Home Admission in the Older Population in Belgium. In Review; 2022 Jan [cited 2022 Mar 3]. Available from: https://www.researchsquare.com/article/rs-1169480/v1 10.1186/s12877-022-03496-4 PMC9585772 36266620

[30] Van der Heyden J, Berete F, Renard F, Vanoverloop J, Devleesschauwer B, De Ridder K (2021). Assessing polypharmacy in the older population: comparison of a self-reported and prescription based method. Pharmacoepidemiol Drug Saf.

[31] Agence InterMutualiste -InterMutualistisch Agentschap (AIM-IMA). Agence InterMutualiste -InterMutualistisch Agentschap. [cited 2021 Jul 26]. Available from: https://www.ima-aim.be/-Donnees-de-sante

[32] Finaba Berete JV, Heyden S, Demarest. Rana Charafeddine. Couplage des données de l’enquête de santé avec les données des organismes assureurs - Hislink 2013 Méthodologie et étude comparative sur la prévalence des maladies chroniques. 2020 Apr.

[33] Regulation (EU) 2016/679 of the European Parliament and of the Council of 27 April. 2016 on the protection of natural persons with regard to the processing of personal data and on the free movement of such data, and repealing Directive 95/46/EC (General Data Protection Regulation). 2016 [cited 2023 Sep 28]. Available from: https://eur-lex.europa.eu/legal-content/EN/TXT/PDF/?uri=CELEX:32016R0679&qid=1695900759326.

[34] GDPR-in-short. European legislation related to data standards. 2021 [cited 2023 Oct 24]. Available from: https://www.polisnetwork.eu/wp-content/uploads/2021/03/GDPR-in-short2.pdf

[35] Harron KL, Doidge JC, Knight HE, Gilbert RE, Goldstein H, Cromwell DA (2017). A guide to evaluating linkage quality for the analysis of linked data. Int J Epidemiol.

[36] Williams N, Hermans K, Stevens T, Hirdes JP, Declercq A, Cohen J (2021). Prognosis does not change the landscape: palliative home care clients experience high rates of pain and nausea, regardless of prognosis. BMC Palliat Care.

[37] Austin PC (2009). Using the standardized difference to compare the prevalence of a Binary Variable between two groups in Observational Research. Commun Stat - Simul Comput.

[38] Saunders NR, Janus M, Porter J, Lu H, Gaskin A, Kalappa G et al. Use of administrative record linkage to measure medical and social risk factors for early developmental vulnerability in Ontario, Canada. IJPDS. 2021 Feb 11 [cited 2022 Mar 3];6(1). Available from: https://ijpds.org/article/view/1407 10.23889/ijpds.v6i1.1407 PMC8107638 34007902

[39] Berete F, Van der Heyden J, Demarest S, Van Oyen H, Charafeddine R, Bruyère O. Effectiveness of protective measures on dental care utilization: analysis from linked database. Eur J Pub Health. 2020;30(5).

[40] Berete F, Charafeddine R, Demarest S, der Heyden JV, Gisle L, Van Den Broucke S et al. Does health literacy mediate the relationship between socioeconomic status and health(-related) outcomes in the Belgian adult population? Will be submitted to BMC Public Health. 2023. 10.1186/s12889-024-18676-7 PMC11055376 38678179

[41] Gorasso V, Moyersoen I, Van der Heyden J, De Ridder K, Vandevijvere S, Vansteelandt S (2022). Health care costs and lost productivity costs related to excess weight in Belgium. BMC Public Health.

[42] Van der Heyden J, Berete F, Devleesschauwer B, De Ridder K, Bruyère O, Renard F (2021). Association between polypharmacy and mortality in the community-dwelling older population: a data linkage study. Int J Epidemiol.

[43] Harron K, Doidge J, Challenges. and opportunities in using administrative data linkage for research: the importance of quality assessment for understanding bias. 2020; UCL Great Ormond Street Institute of Child Health. Available from: https://www.ucl.ac.uk/population-health-sciences/sites/population_health_sciences/files/1-nash-mina_katieharron_jan2020.pdf

[44] Ludvigsson JF, Otterblad-Olausson P, Pettersson BU, Ekbom A (2009). The Swedish personal identity number: possibilities and pitfalls in healthcare and medical research. Eur J Epidemiol.

[45] Dusetzina SB, Tyree S, Meyer AM, Meyer A, Green L, Carpenter WR. Linking data for health services research: a framework and instructional guide. 2014. 25392892

[46] Harron K, Mackay E, Elliot M. An introduction to data linkage. 2016.

[47] Gilbert R, Lafferty R, Hagger-Johnson G, Harron K, Zhang LC, Smith P (2018). GUILD: GUidance for information about linking data sets†. J Public Health.

[48] Bohensky MA, Jolley D, Sundararajan V, Evans S, Pilcher DV, Scott I (2010). Data linkage: a powerful research tool with potential problems. BMC Health Serv Res.

[49] Sediq R, Van Der Schans J, Dotinga A, Alingh RA, Wilffert B, Bos JH et al. Concordance assessment of self-reported medication use in the Netherlands three-generation lifelines Cohort study with the pharmacy database iaDB. Nl: the PharmLines initiative. Clin Epidemiol. 2018;981–9. 10.2147/CLEP.S163037 PMC6101003 30147377

[50] van Brug HE, Rosendaal FR, van Steenbergen LN, Nelissen RG, Gademan MG (2023). Data linkage of two national databases: lessons learned from linking the Dutch Arthroplasty Register with the Dutch Foundation for Pharmaceutical Statistics. PLoS ONE.

[51] Applying for linked data from the Lifelines Cohort Study and IADB.nl database. 2021. Available from: http://wiki-lifelines.web.rug.nl/lib/exe/fetch.php?media=pharmlines_procedures_20210415.pdf

[52] Jutte DP, Roos LL, Brownell MD (2011). Administrative record linkage as a Tool for Public Health Research. Annu Rev Public Health.

[53] European Parliament and European Council. Regulation (EU) 2022/868 of the European Parliament and of the Council of 30 May 2022 on European data governance and amending Regulation (EU) 2018/1724 (Data Governance Act). 2022 [cited 2022 Sep 27]. Available from: https://data.consilium.europa.eu/doc/document/PE-85-2021-INIT/en/pdf

[54] Sakshaug JW, Couper MP, Ofstedal MB, Weir DR (2012). Linking Survey and Administrative records: mechanisms of Consent. Sociol Methods Res.

[55] Sakshaug JW, Schmucker A, Kreuter F, Couper MP, Holtmann L (2021). Respondent understanding of data linkage consent.

[56] van Veen EB (2018). Observational health research in Europe: understanding the General Data Protection Regulation and underlying debate. Eur J Cancer.

[57] Hafferty JD, Campbell AI, Navrady LB, Adams MJ, MacIntyre D, Lawrie SM (2018). Self-reported medication use validated through record linkage to national prescribing data. J Clin Epidemiol.

[58] Richardson K, Kenny RA, Peklar J, Bennett K (2013). Agreement between patient interview data on prescription medication use and pharmacy records in those aged older than 50 years varied by therapeutic group and reporting of indicated health conditions. J Clin Epidemiol.

[59] Rosella LC, Manuel DG, Burchill C, Stukel TA (2011). For the PHIAT-DM team. A population-based risk algorithm for the development of Diabetes: development and validation of the Diabetes Population Risk Tool (DPoRT). J Epidemiol Community Health.

[60] Rosella LC, Fitzpatrick T, Wodchis WP, Calzavara A, Manson H, Goel V (2014). High-cost health care users in Ontario, Canada: demographic, socio-economic, and health status characteristics. BMC Health Serv Res.

[61] Gorman E, Leyland AH, McCartney G, White IR, Katikireddi SV, Rutherford L (2014). Assessing the representativeness of Population-Sampled health surveys through linkage to Administrative Data on Alcohol-related outcomes. Am J Epidemiol.

[62] Meyer BD, Mittag N. Combining administrative and survey data to improve income measurement. Administrative Records for Survey Methodology. 2021;297–322.

[63] Morgan K, Page N, Brown R, Long S, Hewitt G, Del Pozo-Banos M (2020). Sources of potential bias when combining routine data linkage and a national survey of secondary school-aged children: a record linkage study. BMC Med Res Methodol.

[64] Linnenkamp U, Gontscharuk V, Brüne M, Chernyak N, Kvitkina T, Arend W (2020). Using statutory health insurance data to evaluate non-response in a cross-sectional study on depression among patients with Diabetes in Germany. Int J Epidemiol.

[65] Bradley CJ, Penberthy L, Devers KJ, Holden DJ (2010). Health Services Research and Data linkages: issues, methods, and directions for the future: Health Services Research and Data linkages. Health Serv Res.

[66] Maddocks J, Mathieu L, Richards R, Saelaert M, Van Hoof W. tehdas-healthy-data-an-online-citizen-consultation-about-health-data-reuse-intermediatereport.pdf. 2022 Jun [cited 2022 Sep 27]. Available from: https://tehdas.eu/app/uploads/2022/07/tehdas-healthy-data-an-online-citizen-consultation-about-health-data-reuse-intermediate-report.pdf.

[67] Marie Thornby LC. Collecting Multiple Data Linkage Consents in a Mixed-mode Survey: Evidence from a large-scale longitudinal study in the UK. 2018 [cited 2022 Sep 27]; Available from: https://surveyinsights.org/?p=9734

[68] Sakshaug JW, Vicari BJ (2018). Obtaining record linkage consent from establishments: the Impact of Question Placement on Consent Rates and Bias. J Surv Stat Methodol.

[69] Sakshaug JW, Schmucker A, Kreuter F, Couper MP, Singer E (2019). The Effect of Framing and Placement on linkage consent. Pub Opin Q.

[70] European Commission, REGULATION OF THE EUROPEAN PARLIAMENT, AND OF THE COUNCIL on the European Health. Data Space. 2022 [cited 2023 Feb 9]. Available from: https://eur-lex.europa.eu/resource.html?uri=cellar:dbfd8974-cb79-11ec-b6f4-01aa75ed71a1.0001.02/DOC_1&format=PDF.

[71] McLennan S, Celi LA, Buyx A (2020). COVID-19: putting the General Data Protection Regulation to the test. JMIR Public Health Surveill.

[72] Kiseleva A, De Hert P (2021). Creating a European Health Data Space: obstacles in four key legal area. EPLR.

[73] Dibben C, Elliot M, Gowans H, Lightfoot D, Harron K, Dibben C, Goldstein H, Data Linkage Centres (2015). The data linkage environment. Methodological developments in data linkage chap.

[74] Jones KH, Ford DV, Jones C, Dsilva R, Thompson S, Brooks CJ (2014). A case study of the Secure Anonymous Information linkage (SAIL) gateway: a privacy-protecting remote access system for health-related research and evaluation. J Biomed Inform.

[75] Boyd JH, Ferrante AM, O’Keefe CM, Bass AJ, Randall SM, Semmens JB (2012). Data linkage infrastructure for cross-jurisdictional health-related research in Australia. BMC Health Serv Res.

[76] Aldridge RW, Shaji K, Hayward AC, Abubakar I. Accuracy of Probabilistic Linkage Using the Enhanced Matching System for Public Health and Epidemiological Studies. Pacheco AG, editor. PLoS ONE. 2015;10(8):e0136179. 10.1371/journal.pone.0136179 PMC4547731 26302242

[77] Harron K, Goldstein H, Wade A, Muller-Pebody B, Parslow R, Gilbert R, Linkage. Evaluation and Analysis of National Electronic Healthcare Data: Application to Providing Enhanced Blood-Stream Infection Surveillance in Paediatric Intensive Care. Trotter CL, editor. PLoS ONE. 2013;8(12):e85278. 10.1371/journal.pone.0085278 PMC3869925 24376874

