# Supplementary material for: Linking health survey data with health insurance data: methodology, challenges, opportunities and recommendations for public health research. An experience from the HISlink project in Belgium
Source: Arch Public Health. 2023 Nov 15;81:198. doi: 10.1186/s13690-023-01213-0 (PMC10648729; doi:10.1186/s13690-023-01213-0)
Supplement: Supplementary file 1 — Supplementary Material 1 [file 13690_2023_1213_MOESM1_ESM.docx]

**Supplementary data**

Table S1: Overview of the contents of the HISlink 2013 and 2018 databases, Belgium

| **Modules** | **From BHIS source** | **From BCHI source** |
| --- | --- | --- |
|  | **Description / Operationalisation** | **Description / Operationalisation** |
| **Information related to the survey** |  |  |
| ID | Identification number of respondent |  |
| Participated to survey | Status of actual participation to the survey (yes/no) |  |
| Date of interview | Date of interview (DD/MM/YYYY) |  |
| Year of the survey | Year of the survey (YYYY) |  |
| Weight of individual within the sample | Individual post stratification weights |  |
| Availability of self-completed questionnaire | Status of self- competed questionnaire:  - Self-competed questionnaire not required and not available  - Self-competed questionnaire not required, but available  -Self-completed questionnaire required, but not available  -Self-completed questionnaire required and available |  |
| Household cluster | Identification of the household |  |
| **Socio-demographic characteristics** |  |  |
| Age | Age (in years) | Year of birth |
| Sex | Gender (Male / Female) | Gender (Male / Female) |
| Education | Educational attainment based on the highest level of education achieved according to the ISCED 1997 in four categories: No diploma or primary education / Lower secondary / Higher secondary / Higher. |  |
| Place of residence | Province (11 categories) / Region (3 categories) of residence at the moment of the survey | Province (11 categories) of residence at the moment of the survey |
| Household composition / number of persons in the household | Household composition based on reference person in national register. | Health insurance household based on MAF head of household. |
| Nationality/country of birth | Nationality / Country of birth (3 categories): Belgian / Non Belgian – EU / Non-Belgian – non EU. |  |
| Housing | Housing tenure (Owner, co-owner or usufructuary, Renter from an individual private landlord or society, Renter from a social housing association or another public association, Living rent-free). |  |
| Income | The equivalent household income (quintiles based on Belgian population) |  |
| Employment | Current (last) employment / non-employment status | Unemployment status during the last trimester preceding the reference year. |
| Entitlement to increased reimbursement |  | Receipt of a disability or invalidity allowance, take-up and use of increased reimbursement status, maximum billing system, Lump sum for the chronically ill. |
| Insurance status |  | Insurance status of the individual: Undefined situations or no entitlement / Employee (under the general scheme) entitled to large risks / Self-employed person entitled to comprehensive cover". |
| **Health status** |  |  |
| Perceived health | Self-reported indicator based on the question: “How is your health in general?”. Five response categories are possible: Very good / Good / Fair / Poor / Very poor. |  |
| Chronic conditions | Self-reported chronic conditions based on the question: “Have you suffered during the last 12 months from the following disease?” followed by a list of 35 chronic conditions: asthma, chronic bronchitis, chronic obstructive pulmonary disease or emphysema, Parkinson's disease, high blood pressure, epilepsy, myocardial infarction, coronary heart disease, serious heart disease (except myocardial infarction of coronary heart disease), stroke (or consequences), chronic fatigue for a period of at least 3 months, rheumatoid arthritis, osteoarthritis, osteoporosis, diabetes, disorder of the larger or the small bowel for at least 3 months, allergy, serious disease of the kidney other than stones in the kidney, stones in the kidney, stomach ulcer, chronic cystitis, cirrhosis of the liver, liver dysfunction, serious or chronic skin disease, cancer, gallstones of inflammation of the gallbladder, severe headache such as migraine, serious gloom or depression, thyroid problems, high cholesterol level in blood, narrowing of bloodvessels, low back disorder, neck disorder, urinary incontinence, broken hip, prostate problems, eye diseases (diabetic retinopathy, macula degeneration, cataract, glaucoma, other eye disease). | Proxy for diagnostic information (pseudo pathologies) based on the ATC-codes of dispensed medication in public pharmacies, including : cardiovascular disorders, diabetes, asthma, epilepsy, chronic obstructive pulmonary disease, thyroid disorders, cancers, Parkinson’s disease, HIV, cystic fibrosis, exocrine pancreatic diseases, psoriasis, rheumatoid arthritis, psychosis , chronic hepatitis B and C, multiple sclerosis, organ transplantation, Alzheimer's disease, renal failure, hemophilia. |
| Functional limitations | Self-reported functional limitations / restrictions in daily activities due to health problems |  |
| Mental health | Self-reported information in different dimensions of mental health: wellbeing/distress, disorders/symptoms, eating behaviors, suicidal behaviors, positive mental health/vitality, use of psychotropic medicine and self-perceived depression. |  |
| Physical pain | Self-reported bodily pain during the past four weeks. |  |
| Quality of life | Self-reported information on the impact of health status on quality of life, assessed along five dimensions: mobility, personal autonomy, daily activities, pain/discomfort and anxiety/depression. |  |
| Absence from work because of health problems | Self-reported information on absenteeism and number of days absent from work due to health problems. |  |
| Frailty* | Self-reported information on the vulnerability or fragility of the elderly population. |  |
| Children’s strengths and difficulties | Self-reported information on emotional, behavioural and attentional disorders in children and adolescents, and their possible management. |  |
| **Lifestyle and health behavior** |  |  |
| Smoking | Self-reported information on smoking (current smokers, former smokers and non-smokers). |  |
| Use of electronic cigarettes* | Self-reported use of e-cigarettes or similar devices such as electronic chicha, pipes or cigars |  |
| Use of alcohol | Self-reported alcohol consumption. |  |
| Use of illicit drugs | Self-reported use of illicit drugs. |  |
| Physical activity | Self-reported physical activity. |  |
| Nutritional status | Self-reported nutritional status (weight, height) and the resulting body mass index – BMI. |  |
| Nutritional habits | Self-reported eating habits of the population: consumption of fruit, vegetables or salads, 100% pure juices, sweetened drinks, sweet or salty snacks, calcium-enriched dairy products or vegetable products, the amount of water drunk daily, etc. or vegetable products enriched with calcium, the amount of water drunk daily and breakfast (frequency); food allergies or intolerances doctor). |  |
| Dental health | Self-reported information on oral health in the population: use of dental prostheses, frequency of brushing frequency of tooth brushing, limitations caused by oral problems. |  |
| Sexual health | Self-reported information on practices and use of different methods of contraception within the population. |  |
| Gambling* | Self-reported information on gambling addiction problems (casino games, slots, bingo, scratch cards, sports betting, etc.). |  |
| **Health Prevention and attitudes** |  |  |
| Cancer screening | Self-reported information on colorectal cancer, breast cancer and cervical cancer screening, based on the question: “Have you ever had a faecal occult blood test / colonoscopy /mammography / cervical smear test?” and “When was the last you had a faecal occult blood test / colonoscopy /mammography / cervical smear test?”. | Specific nomenclature codes for reimbursement of mammograms performed as part of screening programmes or outside screening programmes. |
| Vaccination | Self-reported vaccination against influenza, pneumococcus and human papillomavirus. | ATC-codes of supplied vaccines |
| Screening for cardiovascular risk factors and diabetes | Self-reported information on methods of preventing cardiovascular disease and diabetes, including control of blood pressure, blood sugar and cholesterol levels. |  |
| Knowledge and attitudes towards HIV | Self-reported information on the knowledge and beliefs of the population on the transmission of the AIDS virus and effective methods of protection against transmission. |  |
| Health literacy* | Self-reported information on the level of health literacy in the population (motivation and skills of individuals to access, understand, evaluate and use information to make decisions about their health). |  |
| **Use of health care and other services** |  |  |
| Contacts with GP | Self-reported information on contacts with GP in the last 12 months. | Specific nomenclature and competence codes for contacts with GPs in outpatient and inpatient settings, date and type of services.. |
| Contacts with specialist | Self-reported information on contacts with specialist in the last 12 months. | Specific nomenclature and competence codes for contacts with specialists in outpatient and inpatient settings, date and type of services. |
| Contacts with emergency department of hospital | Self-reported information on contacts with emergency department of hospital in the last 12 months. | Specific nomenclature codes for contacts with emergency department of hospital, date and type of services |
| Contacts with dentist | Self-reported information on contacts with dentist in the last 12 months. | Specific nomenclature and competence codes for contacts with dentists, date and type of services. |
| Contact with paramedical professionals | Self-reported information on contacts with paramedical professionals in the last 12 months. | Specific nomenclature and competence codes for contacts with paramedical professionals in outpatient and inpatient settings, date and type of services |
| Contact with practitioners from non-conventional medicine | Self-reported information on contacts with practitioners from non-conventional medicine in the last 12 months. |  |
| Contacts with home care services | Self-reported information on the use of home care services in the last 12 months in the event of health problems. These services comprise for example home care services provided by a nurse or midwife, home help for the housework or for older people, "meals on wheels" or transport service. |  |
| Admission to hospital | Self-reported information on hospital admission in the last 12 months. | Specific codes for outpatient and inpatient admission, admission date, discharge date, types of services /procedures. |
| Admission to rest home or nursing home |  | Nomenclature number, service start date, amount (co-payment + reimbursed). |
| Use of medicines | Information on the actual use of medicines, including information on the medicines prescribed or purchased. Thus both non-prescription and prescription medicines are also taken into account. The definition of "medicine" is broader and includes food supplements, medicinal plants, homeopathic products, contraceptive pills, etc. | Prescriptions for reimbursable medicines: CNK code, date of supply, amount (co-payment + reimbursed) |
| Accessibility of health care | Self-reported relative burden of healthcare expenditure on household budgets. |  |
| Patient experiences | Self-reported information on patients experiences from the moment they make an appointment to the consultation and prescription of treatment, in order to obtain an overall assessment of the quality of care services. |  |
| **Physical and social health environment** |  |  |
| Passive smoking | Self-reported passive smoking. |  |
| Other environmental factors affecting health | Self-reported information on the nuisance in the neighborhood or district and the nuisance felt at home (inside the house) and coming from the immediate environment. home (inside the house) and from the immediate environment. |  |
| Accidents | Self-reported information on domestic, road or leisure accidents during leisure time, resulting in injury as well as falls among older population. |  |
| Violence | Self-reported information on interpersonal violence. |  |
| Social health | Self-reported information on the integration into a social network and the support that the person can in the event of a problem. This information involves identifying groups of people who are isolated or socially deprived, and examining the link with physical and mental health. |  |
| Informal care | Self-reported information on informal (non-professional) help given to people with age-related problems or long-term illnesses. The perspective is that of providing help, not receiving it. |  |
| **Mortality data** |  |  |
| Death status |  | Status of death (Yes /No) |
| Date of death |  | Date of death (dd/mm/yyyy) |
| **Health care expenditures** |  |  |
| Amount reimbursed for healthcare use |  | Amount refunded by health insurance. |
| Out-of-pocket |  | Personal intervention. |
| Supplements |  | Additional amount or amount for non-refundable products, services or supplies. |
